# Supplementary material for: Microparticle RSV Vaccines Presenting the G Protein CX3C Chemokine Motif in the Context of TLR Signaling Induce Protective Th1 Immune Responses and Prevent Pulmonary Eosinophilia Post-Challenge
Source: Vaccines (Basel). 2022 Dec 5;10(12):2078. doi: 10.3390/vaccines10122078 (PMC9785538; doi:10.3390/vaccines10122078)
Supplement: Supplementary file 1 [file vaccines-10-02078-s001.zip › vaccines-1982893-Supplementary.pdf]

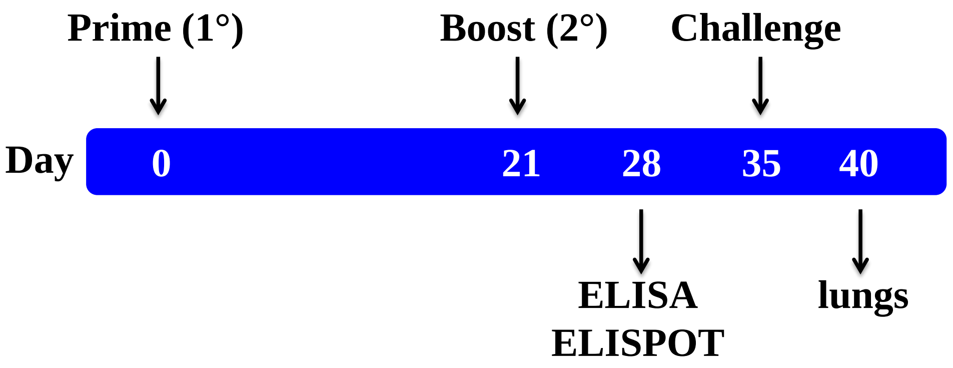

Figure S1. Mouse study regimen. BALB/cJ female mice (6-8 weeks old) were immunized on days 0 and 21 by i.m. administration of LbL-MP suspension or FI-RSV, or on day 0 only by i.n. administration of live RSV A2. Mice were bled on day 28 to obtain sera for ELISA analysis of RSV G-specific antibody responses or sacrificed on day 28 to obtain spleen cells for ELISPOT analysis of RSV G- and M2-specific T-cell responses. Remaining mice were challenged on day 35 by i.n. administration of live RSV A2 and sacrificed on day 40 to obtain lung tissue for analysis of viral burden by plaque assay and BAL fluid for analysis of proteomics by BioLegend LEGENDplex or Luminex and cellomics by flow cytometry.

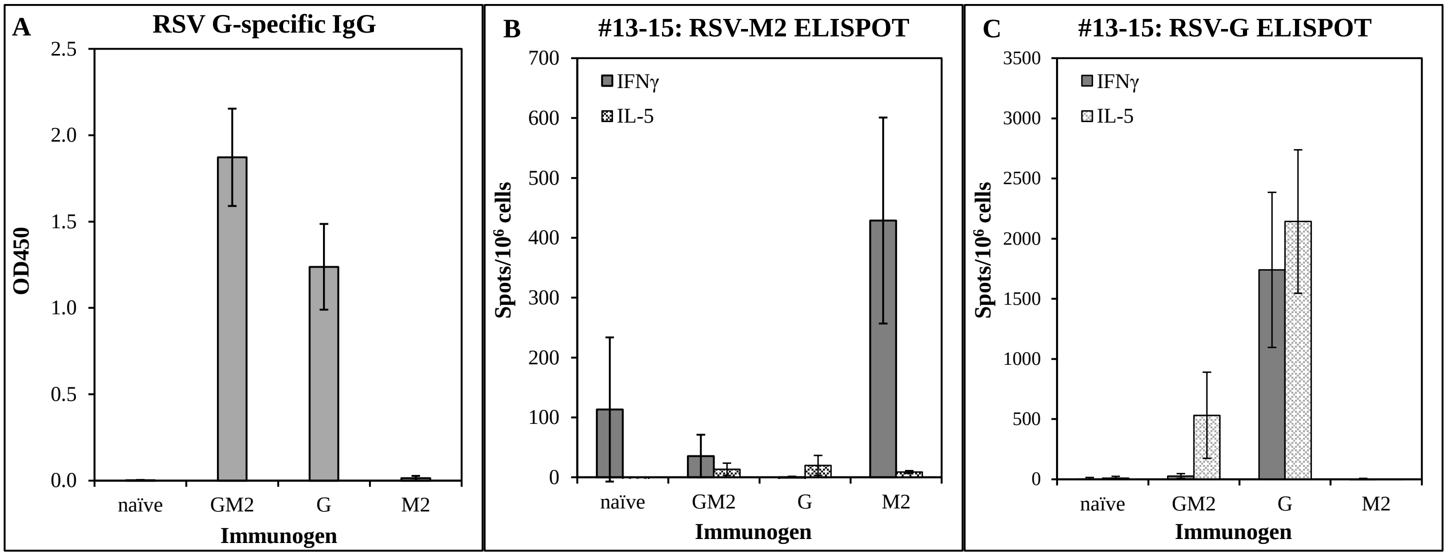

Figure S2. Immune specificity of LbL-MP. BALB/cJ female mice (6-8 weeks old) were immunized on days 0 and 21 by i.m. administration of the indicated LbL-MP suspension. (A) Mice were bled on day 28 and sera were tested in ELISA on RSV G-coated plates. Mean±SEM of 10 mice per group. (B) Mice were sacrificed on day 28 and spleen cells were restimulated with RSV $M2_{81-95}$ peptide. Mean±SEM of 3 mice per group. (C) Day 28 spleen cells were also restimulated with RSV $G_{169-198}$ peptide. Mean±SEM of 3 mice per group.

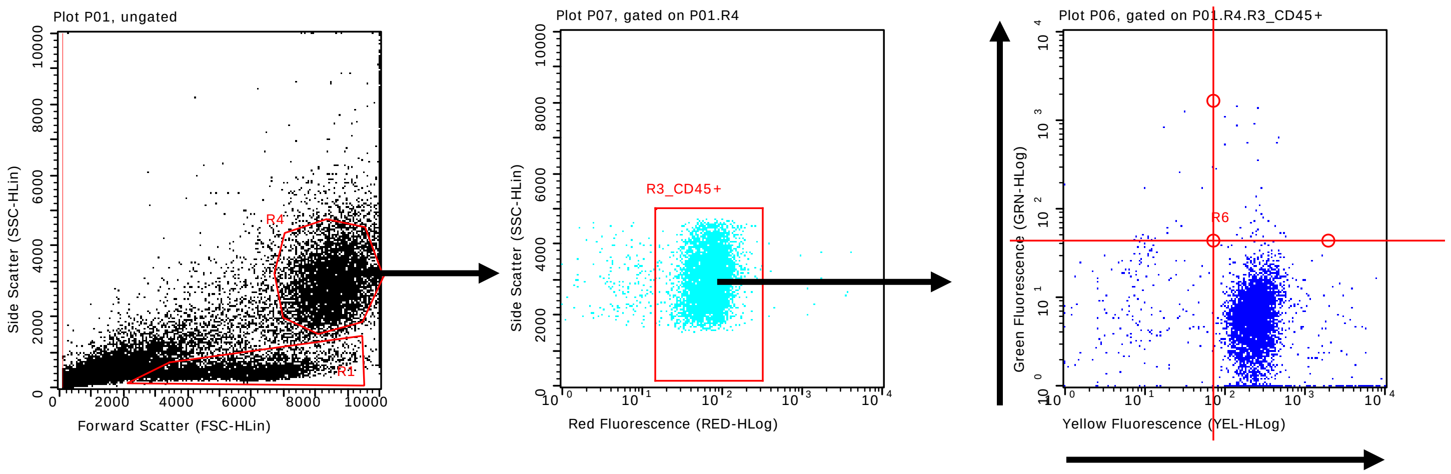

Figure S3. Flow cytometry gating strategy to identify eosinophils. BAL cells were costained with antibodies to CD45, CD11c and SiglecF. Cells were analyzed by flow cytometry and gated by FSC/SSC; R4 population (left) was further gated by CD45 staining (center) and analyzed for CD11c/SiglecF staining (right). Eosinophils were identified as FSC^{high}/SSC^{high}/CD45+/CD11c^{low}/SiglecF^{high}.
